# A method for confirming a third‐party assay of I‐125 seeds used for prostate implants

**DOI:** 10.1002/acm2.12000

**Published:** 2016-11-24

**Authors:** John S. Muryn, D. Allan Wilkinson

**Affiliations:** ^1^ Department of Physics Cleveland State University Cleveland OH USA; ^2^ Department of Radiation Oncology Cleveland Clinic Cleveland OH USA

**Keywords:** brachytherapy, I‐125 seeds, third‐party assay

## Abstract

The purpose of this work is to describe a method and apparatus that can be used to confirm the source strength of a large number of I‐125 seeds while maintaining sterility, accuracy, reproducibility, and time efficiency. Source strengths ranging from 0.395 to 0.504 U/seed were available for this study. Three different seed configurations were measured: loose, linked, and loaded needles. A third‐party 10% assay (NIST traceable) was provided. A custom stand was built out of aluminum to hold an exposure meter [Inovision (Fluke) 451P pressurized ion chamber] at 25 cm above the I‐125 sources to measure the exposure rate. The measurements were made in an operating room, and a sterile sheet was placed under the nonsterile aluminum stand on a sterile loading table. Seeds and needles were placed in a sterile tray for these measurements. Two hundred and six loose seeds in six batches (0.395, 0.395, 0.409, 0.444, 0.444, and 0.444 U/seed) and 1434 seeds in 10 batches containing various strands (0.444, 0.444, 0.444, 0.444, .0444, 0.466, 0.466, 0.504, and 0.504 U/seed) were measured. For the loose and stranded seeds, the average exposure rate per unit activity was measured to be 0.589 mR/h·U with a standard deviation of 0.017. Loaded needles were measured with an average exposure rate per unit activity to be 0.269 mR/h·U with a standard deviation of 0.014. We conclude that the method described here is capable of confirming a third‐party assay when performed on a large number of loose or stranded seeds in bulk. It is less reliable for preloaded needles.

## 1 Introduction

The American Cancer Society estimates that there will be 220,800 new cases of prostate cancer in the US during 2015,^1^ making prostate cancer second in prevalence to skin cancer in men. A widely used treatment method for prostate cancer is the use of radioactive seed implants. In order for the prostate carcinoma to be treated accurately, it is important that the activity of these seeds be known with reasonable precision. There have been many recommendations by American Association of Physicists in Medicine (AAPM) task groups on the accuracy of seed calibration. The AAPM TG‐40,^2^ TG‐56,^3^ and TG‐64^4^ all recommend that at least 10% of the I‐125 seeds have their activity verified against the manufacturers measurements, and if there is a discrepancy above 5% then it needs to be reported to the manufacturer. The task groups do not explicitly specify who should perform the assay of 10% of the seeds. This has led to “third‐party assays”, a practice explicitly allowed by NRC agreement states.

In 2008, the Low Energy Brachytherapy Source Calibration Working Group set out to clarify the recommendations by TG‐40, TG‐50, and TG‐64. This report says “Third party assays do not absolve the institutional physicist from the responsibility to perform the institutional measurement and attest to the strength of the implanted sources”,^5^ The American College of Radiology (ACR) Technical Standard for the Performance of Low‐Dose‐Rate Brachytherapy Physics^6^ also states that each facility should have the instrumentation and methods for verifying source strength in brachytherapy. This does not mean that the third‐party assay should not be used, but rather that the medical physicist is also responsible for verifying the activity of the seeds as defined in the previously cited Task Group reports.

In our practice, both loose and stranded seeds are used roughly in the ratio of 1:5. Both types are supplied sterile and opened in the OR on a sterile loading platform behind a clear lead block shield (Capintec, Inc., Ramsey, NJ) In order for us and others doing OR planning and sterile needle loading, a method is needed for confirming the manufacturer's stated activity. Two general solutions have been proposed. One is to order extra seeds from the same lot as those to be used in the implant and then assay them.^5^ Another way is to assay the seeds in sterile packaging using a calibrated well chamber^7^ or an imaging plate.^8^ Both solutions have limitations. The extra seeds may or may not be representative of those in the implant, especially for the stranded product. The use of the well chamber or imaging plate limits the number of seeds to be assessed (15) at any one time. The purpose of this work is to introduce a method and apparatus that can be used to confirm the source strength of 100% of I‐125 seeds while maintaining sterility, accuracy, reproducibility, and time efficiency. The proposed method is not meant to replace the use of the local 10% assay for those who prefer that approach, but rather to provide confidence in a third‐party assay.

## 2 Materials and methods

The I‐125 seeds used in this study (STM 1251; Bard Medical Division, Covington, GA, USA) were available as loose seeds (50 in a sterile glass vial) or linked in three, four, or five seed configurations (180 in a sterile metal case). Our standing order calls for 0.499 U/seed on the reference date. A third‐party 10% assay (NIST traceable) is provided. Since we bank seeds for future use, source strengths ranging from 0.395 to 0.504 U were available for this study (Table 1).

**Table 1:** The third column, activity per source (U), was calculated based on the current institutional standing order for 0.499 U/seed on a Sunday reference date. The total batch activity (U) was calculated by multiplying the number of sources by the activity per source

Source type	Number of sources	Activity per source (U)	Total batch activity (U)
Stranded	180	0.504	90.72
Stranded	180	0.504	90.72
Stranded	180	0.444	79.92
Stranded	172	0.444	76.368
Stranded	166	0.444	73.704
Stranded	154	0.466	71.764
Stranded	144	0.444	63.936
Stranded	56	0.466	26.096
Stranded	22	0.444	9.768
Loose	64	0.444	28.416
Loose	49	0.444	21.756
Loose	40	0.395	15.8
Loose	24	0.409	9.816
Loose	21	0.444	9.324
Loose	8	0.395	3.16

**Figure 1:**
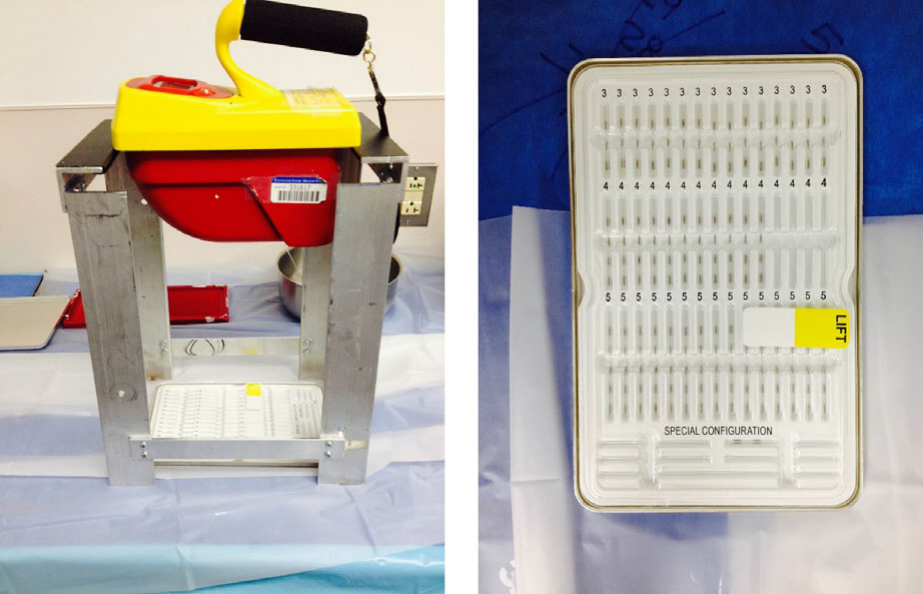
(Left). The exposure meter held by an aluminum stand. The seed tray, seen directly below the exposure meter, can then be slid under the exposure meter for the activity measurement. The clear plastic underneath the stand is a sterile sheet used to separate the nonsterile aluminum stand from the sterile surface of the seed loading table. (Right). The plastic covering over stranded seeds remains intact during the assay to maintain sterility. This is a typical seed configuration for stranded seeds.

**Figure 2:**
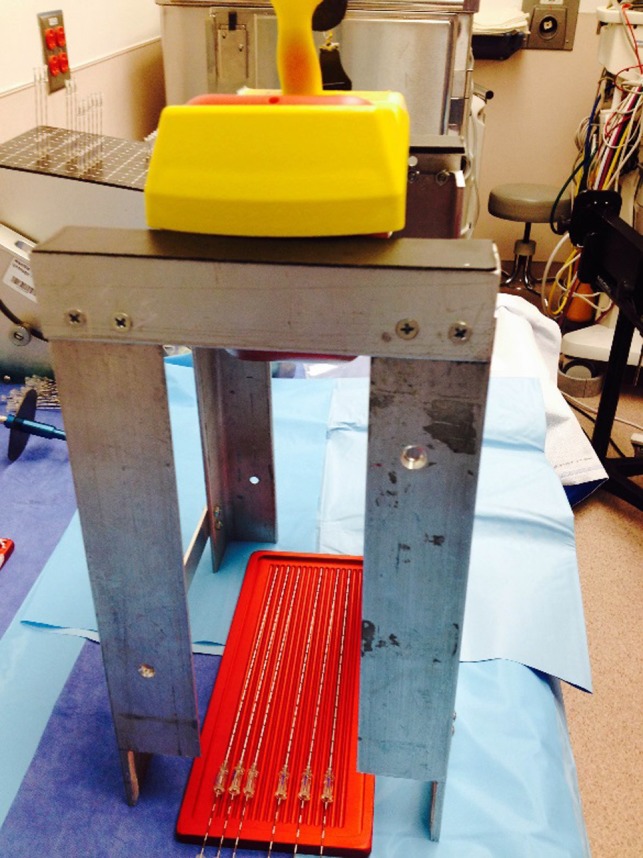
Measurement setup with a tray holding several loaded needles. The modified table legs are visible at one end of the aluminum stand (bottom of the picture).

A custom stand was built out of aluminum to hold an exposure meter (Inovision (Fluke) 451P pressurized ion chamber; Fluke Biomedical, Cleveland, OH, USA) at 25 cm above the I‐125 sources to measure the exposure rate (Fig. 1). The meter is calibrated annually by our Radiation Safety group. We experimented with two different heights, 15 cm and 25 cm. The 25‐cm arrangement was less sensitive to the seed tray position and still easily readable and so was chosen for this work. The height of 25 cm represents the distance from the bottom of the exposure meter to the table surface. We measured three different seed configurations: Loose (6 batches), linked (9 batches), and loaded needles (10 batches) (Bard FastFil Seed Implant Needle). This made a total of 206 loose seeds and 1424 stranded seeds that were assayed, and a total of 224 seeds in loaded needles that were assayed. The stranded seeds are packaged by the manufacturer in a rectangle metal tray containing 180 seeds (Fig. 1). For these measurements, the metal lid was removed, but the plastic cover that maintains sterility was left in place. Loose seeds were placed on a sterile loading tray in no particular configuration for assay, but arranged so as not to produce self‐shielding. Loaded needles were also measured in a sterile tray (Fig. 2). The legs of one side of the stand were machined so that the tray holding either the loose seeds, stranded seeds, or loaded needles could only slid under the exposure meter from one side and stopping them from sliding in too far. The tray position under the meter was adjusted slightly until a maximum reading was observed. Readings stabilized within 2–3 s and typically only three or four tray positions were required to find the maximum value.

One of the goals of this study was to be able to measure the activity of the seeds in the operating room while maintaining the sterility of the seeds. In order to maintain sterility of the seeds as well as the needle‐loading table, a sterile sheet was used under the nonsterile aluminum stand (Fig. 1). Seeds and needles were placed in a sterile tray for these measurements (Fig. 1).

Because intraoperative treatment planning is used in our institution, the loose and stranded seed activity can be measured and verified before needle loading. However, some institutions use a preplan technique, and then order preloaded needles based on their preplan. Because of this, we also measured loaded needles in order to assess the usefulness of this method (Fig. 2). As previously mentioned, the loaded needles were placed onto a sterile tray, but otherwise the activity of the seeds in the needles were assayed in the same way as the loose and stranded seeds (Fig. 2). Various needles with differing seeds per needle were measured at the same time to determine if this had any effect on the seed measurement accuracy (Table 2).

**Table 2:** The third column, activity per source (U), was calculated based on the current institutional standing order for 0.499 U/seed on a reference date. The total batch activity (U) was calculated by multiplying the number of sources by the activity per source

Source type	Seeds per needle	Number of needles	Number of sources	Activity per source (U)	Total activity (U)
Loaded needles	5	6	30	0.444	13.32
Loaded needles	5	6	30	0.466	13.98
Loaded needles	4, 5^a^	3, 3^a^	27	0.466	12.582
Loaded needles	6	4	24	0.444	10.656
Loaded needles	4	6	24	0.466	11.184
Loaded needles	2, 2^a^	5, 6^a^	22	0.444	9.768
Loaded needles	3, 4^a^	3, 3^a^	21	0.466	9.786
Loaded needles	3	6	18	0.466	8.388
Loaded needles	4	4	16	0.444	7.104
Loaded needles	3	4	12	0.444	5.328

a Measurements were made on needles with varying amounts of loaded seeds. For example, in the third column there were three needles with four seeds per needle and three needles with five seeds per needles that were assayed at the same time using the sterile tray.

The measured exposure rate in mR per hour was corrected using the calibration factor from Radiation Safety. The total activity was simply the product of the number of seeds in the measuring tray and the average decayed activity per seed from the third‐party assay.

The whole process of setting up the aluminum stand with the exposure meter on a sterile sheet takes only a few minutes. Exposure measurements can be done in 20–30 sec per tray of seeds. In most instances, we performed the measurements in between cases while the room was being cleaned and prepared so as not to delay the next implant procedure.

## 3 Results

### 3.A Loose and stranded seeds

**Figure 3:**
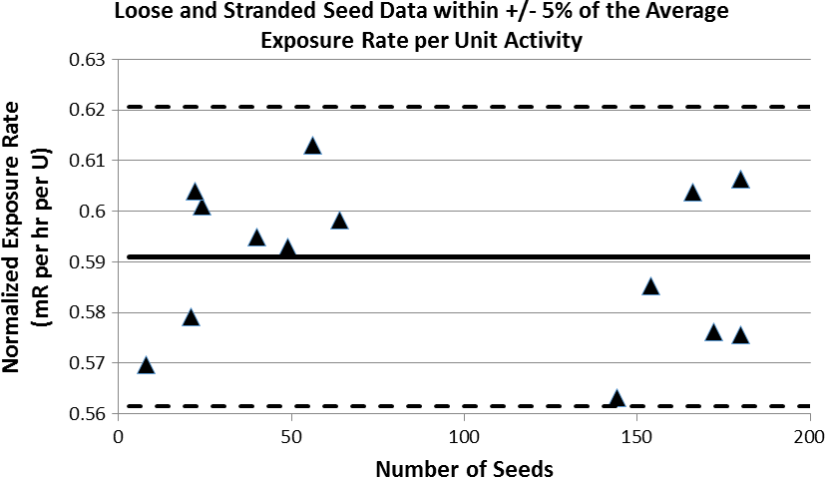
The normalized exposure rate is plotted for all measurements made on loose and stranded seed batches. The two dashed lines represent the 5% difference from the average normalized exposure rate (thick black line).

The average normalized exposure rate for the loose seeds was 0.589 ± 0.012 and stranded seed data, 0.589 ± 0.019 (Table 3) with a combined mean value of 0.589 ± 0.017 mR/h·U. All the measured data fall within ± 5% of the average normalized exposure rate (Fig. 3). According to the AAPM TG‐64 recommendations, a deviation greater than 5% from the manufacturer's specified seed activity should lead to contacting the manufacturer about the difference.

**Table 3:** Column four contains the reading from the exposure meter, and from this measurement the normalized exposure rate was calculated (column five)

Source type	Number of sources	Activity per source (U)	Reading (mR/h)	Normalized exposure rate (mR/h·U)
Stranded	180	0.504	55	0.606
Stranded	180	0.504	55	0.606
Stranded	180	0.444	46	0.576
Stranded	172	0.444	44	0.576
Stranded	166	0.444	44.5	0.604
Stranded	154	0.466	42	0.585
Stranded	144	0.444	36	0.563
Stranded	56	0.466	16	0.613
Stranded	22	0.444	5.9	0.604
Loose	64	0.444	17	0.598
Loose	49	0.444	12.9	0.593
Loose	40	0.395	9.4	0.595
Loose	24	0.409	5.9	0.601
Loose	21	0.444	5.4	0.579
Loose	8	0.395	1.8	0.570

### 3.B Loaded needles

**Figure 4:**
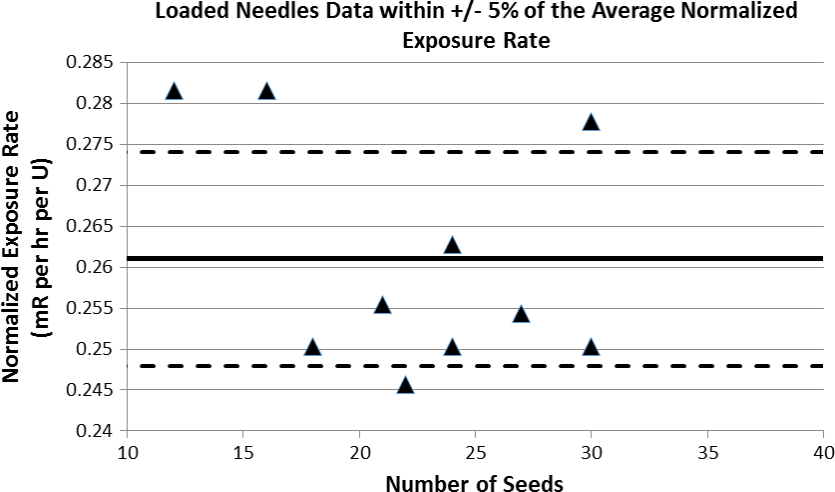
The normalized exposure rate is plotted for all measurements made on loaded needle batches. The two dashed lines represent the 5% difference from the average normalized exposure rate (thick black line).

The average normalized exposure rate for the loose and stranded seed data (Table 4) is 0.263 mR/h·U with a standard deviation of 0.014. For the loaded needles, only 6 of the 10 normalized exposure rate measurements fall within 5% of the average normalized exposure rate (Fig. 4).

**Table 4:** Column four contains the reading from the exposure meter, and from this measurement the normalized exposure rate was calculated (column five)

Source type	Number of sources	Activity per source (U)	Reading (mR/h)	Normalized exposure rate (mR/h·U)
Loaded needles	30	0.444	3.7	0.278
Loaded needles	30	0.466	3.5	0.282
Loaded needles	27	0.466	3.2	0.282
Loaded needles	24	0.444	2.8	0.250
Loaded needles	24	0.466	2.8	0.255
Loaded needles	22	0.444	2.4	0.250
Loaded needles	21	0.466	2.5	0.254
Loaded needles	18	0.466	2.1	0.250
Loaded needles	16	0.444	2	0.263
Loaded needles	12	0.444	1.5	0.246

## 4 Discussion

Ideally, 100% of the radioactive sources used in an implant should be assayed by the end user. However, due to the large number of seeds typically implanted for prostate cancer, a compromise of 10% has been deemed acceptable. The 10% assay may be performed legally by a third party. Besides the obvious convenience, this also provides measurements of seeds from the same batch as are used in manufacturing the stranded product. Nevertheless, good practice demands that the end user confirm the source activity. We are proposing a method that does that for 100% of the sources, in a timely way, under sterile conditions, for both loose and stranded sources without repeating the third‐party seed by seed dosimetry.

The method we are proposing was designed for a busy practice like ours where confirming measurements could be made in the operating room. Sterility is maintained so that the assay can be done in a procedure/operating room. Equipment setup time is short (2 or 3 min) and the measurements take only a few seconds each. Various seed configurations can be easily accommodated. We recognize that the sensitivity of the method (5%) might be of concern. However, in our experience, assaying single low‐activity seeds used for prostate implants with a calibrated system consisting of a Standard Imaging HDR Plus well chamber and a CNMC K602 electrometer operating on the most sensitive scale has a comparable uncertainty. Methods for a bulk assay of seeds using a well chamber have been described. An early report^9^ found that the 100% bulk assay was irreproducible and often erroneous. A five‐seed assay worked much better yielding uncertainties around 5% similar to our results. A more recent study^7^ showed that with proper design and holders, a 15‐seed assay could be performed under sterile conditions and with a 3% uncertainty. The seeds were supplied in a sterile cartridge placed inside a sterile bag for measurement in the well chamber. Another study^8^ used an imaging plate for quality assurance of loaded cartridges. Both methods may be limited to seeds in a cartridge without considerable modification to maintain sterility. Both require much more time for a 100% assay than does our method. However, they were both capable of detecting dead or aberrant sources, whereas our method is not. Incidentally, a 10% assay presumably would miss such a seed 90% of the time. It needs to be pointed out that the manufacturer of the seeds used in this study measures all the seeds for purposes of sorting them into activity bins and so it is unlikely that a dead seed or seeds would evade detection. What seems more likely is some sort of human error in which different seed orders get mixed up or mislabeled. That type of error should be detectable in our batch assay. The question of how to proceed if the measurement indicated a deviation beyond 5% has been discussed.^5^ A decision by the authorized physician user, informed by the uncertainty in the measurement, to continue or not with that particular batch of seeds would need to be made.

One concern in using this method is the exposure to the personnel who are making the measurements. The maximum exposure rate we measured was 55 mR/h at a distance of 25 cm. The meter is approximately 20 cm high and allowing for a few centimeters beyond that for the reading position, the exposure rate at that point should be no greater than 55/4 or 14 mR/h. Allowing 20 s to finalize a reading leads to an exposure of 14/180 or 0.08 mR. Thus, in order to exceed the recommended limit of 2 mR in any hour, the physicist doing the measurement would have to assay roughly 20 batches in the hour. This is highly unlikely, but nevertheless should be kept in mind.

The methodology described here should be easily adaptable to other users since the equipment is either normally found in radiation oncology departments or can be easily fabricated (stand). However, the measurements obtained would depend slightly upon the seed model, but mostly upon the meter used, the geometry of the setup, and the details of the seed holder. We hope that our data would provide a basis for determining the reasonableness of other institutions’ measurements.

## 5 Conclusion

The proposed in‐house batch assay is a reasonable way to verify a third‐party seed calibration for 100% of loose and stranded seeds. The larger uncertainty estimate for preloaded needles, likely related to the considerable photon attenuation of the stainless steel wall, makes this method as described less reliable, although decreasing the distance between needles and meter would increase the exposure rate and possibly overcome this drawback.
